# Antibodies to heat shock protein 90 in osteosarcoma patients correlate with response to neoadjuvant chemotherapy

**DOI:** 10.1054/bjoc.1999.0881

**Published:** 1999-12-08

**Authors:** K Trieb, R Gerth, G Holzer, J G Grohs, P Berger, R Kotz

**Affiliations:** Department of Orthopedics, University of Vienna, Währingergürtel 18-20, Vienna, A-1090, Austria; Institute for Biomedical Aging Research of the Austrian Academy of Sciences, Innsbruck, Austria

**Keywords:** osteosarcoma, heat shock protein 90, antibody, human, ELISA

## Abstract

Autoantibodies to the heat shock protein 90 (Hsp 90) have been reported as prognostic marker in breast cancer patients. Sera from 20 high-grade osteosarcoma patients were tested at the time of diagnosis by enzyme-linked immunosorbent assay. Presence of anti-Hsp90 antibodies correlated with a better response to neoadjuvant chemotherapy (*P*< 0.01), whereas the absence correlated with development of metastases. These data suggest that anti-Hsp90 antibodies might be of predictive value in human osteosarcoma. © 2000 Cancer Research Campaign


British Journal of Cancer (2000) 82(1), 85–87
© 2000 Cancer Research Campaign
Article no. bjoc.1999.0881

Short Communication

### Antibodies to heat shock protein 90 in osteosarcoma patients correlate with response to neoadjuvant chemotherapy

**K Trieb[1], R Gerth[2], G Holzer[1], JG Grohs[1], P Berger[2] and R Kotz[1]**

[1]Department of Orthopedics, University of Vienna, Währingergürtel 18-20, A-1090 Vienna, Austria, [2]Institute for Biomedical Aging Research of the Austrian Academy of Sciences, Innsbruck, Austria

**Summary** Autoantibodies to the heat shock protein 90 (Hsp 90) have been reported as prognostic marker in breast cancer patients. Sera from 20 high-grade osteosarcoma patients were tested at the time of diagnosis by enzyme-linked immunosorbent assay. Presence of anti-Hsp90 antibodies correlated with a better response to neoadjuvant chemotherapy ($P < 0.01$), whereas the absence correlated with development of metastases. These data suggest that anti-Hsp90 antibodies might be of predictive value in human osteosarcoma. © 2000 Cancer Research Campaign

**Keywords**: osteosarcoma; heat shock protein 90; antibody; human; ELISA

Autoantibodies to heat shock protein 90 (Hsp90) in breast cancer patients have been reported to correlate with survival, the presence of involved lymph nodes and metastatic occurrence (Jameel et al, 1993; Conroy et al, 1995; Conroy et al, 1998). Heat shock proteins, which are highly conserved immunogenic proteins performing intracellular chaperoning functions and preventing cells from death, have been shown to be involved in tumour immunity (Lindquist, 1986; Kaufmann, 1990; Suto et al, 1995). In osteosarcoma cell lines, Hsp72 has been shown to be selectively expressed on the cell surface and to be a target for natural killer cells (Multhoff et al, 1997). It has been reported that Hsp27 is overexpressed in human osteosarcomas (Uozaki et al, 1997) and that Hsp72 is de novo expressed (Trieb et al, 1998). The Hsp72 de novo expression correlates with a good response to neoadjuvant chemotherapy and with Hsp60 expression. Additionally, T lymphocytes specific for Hsps were isolated from human osteosarcomas (Trieb et al, 1997). In osteosarcoma histological response to chemotherapy is – in combination with initial tumour size – the most important and generally accepted prognostic factor (Bentzen et al, 1988; Davis et al, 1994; Bieling et al, 1996). But so far, diagnosis is limited to clinical symptoms, radiology and histology; diagnostic laboratory tests are not available. Because anti-Hsp antibodies have not been investigated in human osteosarcoma, it is the aim of the study to determine anti-Hsp90 antibodies in sera from osteosarcoma patients and correlate them with clinical features.

*Received 25 November 1998*
*Revised 1 July 1999*
*Accepted 8 July 1999*

*Correspondence to:* K Trieb

### MATERIALS AND METHODS

### Patients

Sera from 20 patients (ten female, ten male, mean age: 18.6 years, range 9–43) with high-grade osteosarcoma at the time of diagnosis before biopsy and chemotherapy were analysed. The tumour was located in the femur in 15 patients, in the tibia in four patients and in the ilium in one patient. The mean duration of follow-up was 4.3 years (range 0.75–10 years). Informed consent was obtained from the patients included in this study. Immediately following diagnosis, i.e. biopsy, all osteosarcoma patients then received full neoadjuvant multiagent chemotherapy according to a protocol of the German/Austrian/Swiss Cooperative Osteosarcoma Study trial (Bieling et al, 1996). Surgical margins were defined histologically according to the criteria of Enneking et al (1980) and were wide in all cases except one marginal resection in a patient with lung metastases.

Histologic analysis was done on the surgically resected tumours with regard to tumour response to preoperative chemotherapy according to the criteria of Salzer-Kuntschik (grade I–VI; Salzer-Kuntschik et al, 1983). Response to preoperative chemotherapy was considered as good when no or less than 10% viable tumour cells (grade I–III) were found, whereas response was considered as poor (grade IV–VI) when >10% viable tumour cells were found or no effect of chemotherapy was seen.

### Enzyme-linked immunosorbent assay

Antibody titres were tested by a solid-phase-bound antigen indirect enzyme-linked immunosorbent assay (ELISA) as described previously. Shortly, ELISA plates (Maxisorp, Nunc, Rosklide, Denmark), precoated with 120 μl well$^{-1}$ 0.2% glutaraldehyde (Sigma, Deisenhofen, Germany) were coated with 100 μl well$^{-1}$ human Hsp90 (StressGen, Victoria, Canada, 5 μg ml$^{-1}$) in

**Table 1** Anti-hsp90 antibodies in sera of osteosarcoma patients ($n$ = 20) in correlation with response to neoadjuvant chemotherapy and appearance of metastases

| | Osteosarcoma patients | | |
|---|---|---|---|
| | Hsp90-positive ($n$=8) | Hsp90-negative ($n$=12) | $P$-value[a] |
| Response | | | < 0.05 |
| Poor | 2 (25%) | 9 (75%) | |
| Good | 6 (75%) | 3 (25%) | |
| Metastases | | | |
| Yes | 0 (0%) | 3 (25%) | |
| No | 8 (100%) | 9 (75%) | |

[a] Sera for Hsp90 antibodies positive vs. negative (Mann–Whitney $U$-test).

phosphate-buffered saline (PBS) (pH 7.2) and 0.1 M ethanolamine in PBS 100 µl well$^{-1}$ overnight at 4°C. Remaining solid phase binding capacity was blocked (1 h at 37°C) by using 200 µl well$^{-1}$ of 0.1 M ethanolamine in PBS (pH 7.2). After washing, serum samples (100 µl well$^{-1}$, diluted 1:100–500, 90 min, 37°C) were tested for their antibody titres against Hsp90 by peroxidase-conjugated rabbit immunoglobulins to human immunoglobulins and subsequently developed by ABTS. Extinction was read at 410 nm in an automatic ELISA reader (Dynatech, Santa Monica, CA, USA), antibody titres were given as relative units ml$^{-1}$ (U ml$^{-1}$) after subtraction of non-specific binding assessed with ethanolamine. Specificity testing was performed in competitive one-site indirect ELISAs. All densities were quantified without knowledge of serum identity, i.e. blindly.

### Statistical analysis

The association between anti-Hsp titres and clinical parameters was estimated by the Mann–Whitney $U$-test. The significance level was set at a value of $P < 0.05$. All values are given as mean ± S.D. and all tests were performed at least in duplicate.

### RESULTS

Antibodies to Hsp90 could be detected in eight patients suffering from osteosarcoma (8/20, 40%), the mean ± S.D. optical density (OD) level was 0.06 ± 0.03 U ml$^{-1}$ (range: 0.05–0.2). The presence of anti-Hsp90 antibodies correlated with a good response to neoadjuvant chemotherapy. Seventy-five per cent of the patients with a positive anti-Hsp90 antibody titre had a good response to neoadjuvant chemotherapy. In contrast, only 25% of the patients with anti-Hsp90 negative sera had a good response ($P < 0.05$, Table 1).

Although only three patients exhibited metastases, a positive correlation was found between the absence of anti-Hsp90 serum antibodies and lung metastases at the time of diagnosis. All patients with metastases were negative for anti-Hsp90 antibodies, whereas no metastases were detected in patients with a positive anti-Hsp90 titre (Table 1).

When anti-Hsp90 antibodies in osteosarcoma patients were correlated with initial tumour size, duration of symptoms, alkaline phosphatase serum levels, gender or age, no correlation was found (data not shown).

### DISCUSSION

The diagnosis of osteosarcoma, the most frequent malignant bone tumour, is still limited to clinic symptoms, radiology and histology, but so far, an undisputed serological marker is not available for the prognosis of osteosarcoma, although a variety of markers has been investigated. For instance the prognostic value of alkaline phosphatase (AP) and lactate dehydrogenase (LDH) serum levels are controversial. Some groups reported a correlation of pretreatment levels within the normal range of the two enzymes with a better outcome (Bacci et al, 1996), whereas others could only find a correlation for LDH, but not for AP (Pochanugool et al, 1997). A correlation only in patients without metastases, but not in such with metastases has been reported (Meyers et al, 1993). Other serum factors like erythrocyte sedimentation rate were found to be not of prognostic value. Tumour size and histologic response to preoperative chemotherapy have been shown to be the most important prognostic factors, whereas age, gender or localization are of minor prognostic value (Bentzen et al, 1988; Bieling et al, 1996; Pochanugool et al, 1997; Saeteret al, 1997).

In contrast to serum, in tumour tissue several prognostic markers have been identified, for instance $P$-glycoprotein, ErbB-2, genetic aberrations or Hsp72 (Baldini et al, 1995; Onda et al, 1995; Trieb et al, 1998; Tarkkanen et al, 1999). In breast cancer it has been shown that the overexpression of Hsps in the tumour must not correlate with serum antibody levels (Conroy et al, 1995). The immune response against Hsps must not necessarily a general, uniform cellular and humeral response. Autoantibodies against Hsp90 in breast cancer patients have been reported to correlate with survival, the presence of involved lymph nodes and metastatic occurrence (Jameel et al, 1993; Conroy et al, 1995, 1996, 1998). It was, therefore, an aim of this study to investigate whether anti-Hsp antibodies could serve as a predictive marker at the time of diagnosis, because non-response to chemotherapy is one of the main problems in the treatment of osteosarcoma. Our results suggest that the humoural immune response to Hsp90 might be of predictive value, because the presence of anti-Hsp90 antibodies correlates with a good response to neoadjuvant chemotherapy and the absence of anti-Hsp90 antibodies correlates with occurrence of metastases.

The mechanism of the protective effect of Hsp90 is not yet clarified, it might be accompanied by an immune response to the associated Hsp or by a direct protective effect of anti-Hsp90 antibodies. Transfection of tumours with Hsps in vivo resulted in the rejection of the tumour by development of an immune response and tumour cells transfected with Hsps in vitro lost tumorigenicity as compared to untransfected cells (Lukacs at al, 1997).

These observations should stimulate evaluation of the role of Hsp90 in drug resistance in osteosarcoma. After testing a representative sample of sera from patients suffering from osteosarcoma, an entity with a low incidence, we found that anti-Hsp90 antibodies could, among others, act as new predictive markers in osteosarcoma. To further confirm our findings, a larger number of patients needs to be studied.

### ACKNOWLEDGEMENTS

This work was supported by a grant from the Jubiläumsfonds of the Austrian Nationalbank (KT, project no. 6803). The authors thank Mrs Hitchmann for correction of the manuscript.

© 2000 Cancer Research Campaign

**REFERENCES**

Bacci G, Ferrari S, Picci P, Zolezzi C, Gherlinzoni F, Iantorno D and Cazzola A (1996) Methotrexate serum concentration and histological response to multiagent primary chemotherapy for osteosarcoma of the limbs. *J Chemother* **8**: 472–478

Baldini N, Scotlandi K, Barbanti-Bròdano G, Manara M, Maurici D, Bacci G, Bertoni F, Picci P, Sottili S, Campanacci M and Serra M (1995) Expression of p-glycoprotein in high-grade osteosarcomas in relation to clinical outcome. *N Engl J Med* **333**: 1380–1385

Bentzen SM, Poulsen HS, Kaae S, Jensen OM, Johanson H, Mouridsen HT, Daugaard S and Arnoldi C (1988) Prognostic factors in osteosarcomas: a regression analysis. *Cancer* **62**: 194–202

Bieling B, Rehan N, Winkler P, Helmke K, Maas R, Fuchs N, Bielak S, Heise U, Jürgens H, Treuner J, Romanowski R, Exner U, Kotz R and Winkler K (1996) Tumor size and prognosis in aggressively treated osteosarcoma. *J Clin Oncol* **14**: 848–858

Conroy SE and Latchman DS (1996) Do heat shock proteins have a role in breast cancer? *Br J Cancer* **74**: 717–721

Conroy SE, Gibson SL, Brunström G, Isenberg DA, Luqmani Y and Latchman DS (1995) Antibodies to the 90 kD heat shock protein in sera of breast cancer patients? *Lancet* **345**: 126

Conroy SE, Sasieni PD, Fentiman I and Latchman DS (1998) Autoantibodies to the 90 kD heat shock protein and poor survival in breast cancer patients. *Eur J Cancer* **34**: 942–943

Davis AM, Bell RS and Goodwin PJ (1994) Prognostic factors in osteosarcoma: a critical review. *J Clin Oncol* **12**: 423–431

Enneking WF, Spanier SS and Goodman MA (1980) A system for the surgical staging of musculoskeletal sarcoma. *Clin Orthop* **153**: 106–120

Gottesman MM and Pastan I (1993) Biochemistry of multidrug resistance mediated by the multidrug transporter. *Annu Rev Biochem* **62**: 385–427

Jameel A, Law M, Coombes RC and Luqmani YA (1993) Significance of heat shock protein 90 as a prognostic indicator in breast cancer. *Int J Oncol* **2**: 1075–1080

Kaufmann S (1990) Heat shock proteins and the immune response. *Immunol Today* **11**: 129–133

Lindquist S (1986) The heat-shock response. *Ann Rev Biochem* **55**: 1151–1191

Lukacs KV, Nakakes A, Atkins CJ, Lowrie DB and Colston MJ (1997) In vivo gene therapy of malignant tumours with heat shock protein 65 gene. *Gene Ther* **4**: 346–350

Meyers PA, Heller G, Healy JH, Huvos A, Applewhite A, Sun M and LaQuaglia M (1993) Osteogenic sarcoma with clinically detectable metastasis at initial presentation. *J Clin Oncol* **11**: 449–453

Multhoff G, Botzler C, Jennen L, Schmidt J, Ellwart J and Issels R (1997) Heat shock protein 72 on tumor cells. *J Immunol* **158**: 4341–4350

Onda M, Matsuda S, Higaki S, Iijima T, Fukushima J, Yokokura A, Kojima T, Horiuchi H, Kurokawa T and Yamamoto T (1996) ErbB-2 expression is correlated with poor prognosis for patients with osteosarcoma. *Cancer* **77**: 71–78

Pochanugool L, Subhadharaphandou T, Dhanachai M, Hathirat P, Sangthawan D, Pirabul R, Suporn Onsanit S and Pornpipatpong N (1997) Prognostic factors among 130 patients with osteosarcoma. *Clin Orthop Rel Res* **345**: 206–214

Saeter G, Elomaa I, Wahlquist Y, Alvegård TA, Wiebe T, Monge O, Forrestier E and Solheim ØP. Prognostic factors in sarcomas. *Acta Orthop Scand* **68**: 156–160

Salzer-Kuntschik M, Brand G and Delling G (1983) Bestimmung des morphologischen Regressionsgrades nach Chemotherapie bei malignen Knochentumoren. *Pathologe* **4**: 135–141

Suto R and Srivastava P (1995) A mechanism for specific immunogenicity of heat shock protein-chaperoned peptides. *Science* **269**: 1585–1588

Tarkkanen M, Elomaa I, Blomqvist C, Kivioja A, Kellokumpu-Lehtinen P, Böhling T, Valle J and Knuutila S (1999) DNA sequence copy number increase at 8q: a potential new prognostic marker in high-grade osteosarcoma. *Int J Cancer* **84**: 114–121

Trieb K, Windhager R, Lang S, Dirnhofer S and Kotz R (1997) Human osteosarcoma infiltrating lymphocytes recognize the heat shock protein 72. *Trans Orthop Res Soc* **22**: 561

Trieb K, Lechleitner T, Lang S, Windhager R, Kotz R and Dirnhofer S (1998) Heat shock protein 72 expression in osteosarcomas correlates with good response to neoadjuvant chemotherapy. *Hum Pathol* **10**: 1050–1055

Uozaki H, Horiuchi H, Ishida T, Jijima T, Imamura T and Machinami R (1997) Overexpression of metallothioneins, gluthatione-S-transferase, heat shock protein 27 and lung-related protein in osteosarcoma. *Cancer* **79**: 2336–2344

© 2000 Cancer Research Campaign

